# Molecular imaging of glioblastoma multiforme using anti-insulin-like growth factor-binding protein-7 single-domain antibodies

**DOI:** 10.1038/sj.bjc.6605937

**Published:** 2010-10-19

**Authors:** U Iqbal, H Albaghdadi, Y Luo, M Arbabi, C Desvaux, T Veres, D Stanimirovic, A Abulrob

**Affiliations:** 1 Institute for Biological Sciences, National Research Council of Canada, 1200 Montreal Road, Ottawa, Ontario, Canada K1A 0R6; 2 Department of Cellular and Molecular Medicine, Faculty of Medicine, University of Ottawa, 451 Smyth Road, Ottawa, Ontario, Canada K1H 8M5; 3 Department of Environmental Biology, University of Guelph, Guelph, Ontario, Canada N1G 2W1; 4 Industrial Materials Institute, National Research Council of Canada, 75 de Mortagne Boulevard, Boucherville, Quebec, Canada J4B 6Y4

**Keywords:** molecular imaging, insulin-like growth factor-binding protein 7, single-domain antibody, brain cancer, glioblastoma, nanoparticles

## Abstract

**Background:**

Insulin-like growth factor-binding protein 7 (IGFBP7) is an abundant, selective and accessible biomarker of glioblastoma multiforme (GBM) tumour vessels. In this study, an anti-IGFBP7 single-domain antibody (sdAb) was developed to target GBM vessels for molecular imaging applications.

**Methods:**

Human GBM was modelled in mice by intracranial implantation of U87MG.EGFRvIII cells. An anti-IGFBP7 sdAb, isolated from an immune llama library by panning, was assessed *in vitro* for its binding affinity using surface plasmon resonance and by *ex vivo* immunobinding on mouse and human GBM tissue. Tumour targeting by Cy5.5-labelled anti-IGFBP7 sdAb as well as by anti-IGFBP7 sdAb conjugated to PEGylated Fe_3_O_4_ nanoparticles (NPs)-Cy5.5 were assessed in U87MG.EGFRvIII tumour-bearing mice *in vivo* using optical imaging and in brain sections using fluorescent microscopy.

**Results:**

Surface plasmon resonance analyses revealed a medium affinity (*K*_D_=40–50 nM) binding of the anti-IGFBP7 sdAb to the purified antigen. The anti-IGFBP7 sdAb also selectively bound to both mouse and human GBM vessels, but not normal brain vessels in tissue sections. *In vivo*, intravenously injected anti-IGFBP7 sdAb-Cy5.5 bound to GBM vessels creating high imaging signal in the intracranial tumour. Similarly, the anti-IGFBP7 sdAb-functionalised PEGylated Fe_3_O_4_ NP-Cy5.5 demonstrated enhanced tumour signal compared with non-targeted NPs. Fluorescent microscopy confirmed the presence of anti-IGFBP7 sdAb and anti-IGFBP7 sdAb-PEGylated Fe_3_O_4_ NPs selectively in GBM vessels.

**Conclusions:**

Anti-IGFBP7 sdAbs are novel GBM vessel-targeting moieties suitable for molecular imaging.

Glioblastoma multiforme (GBM) is a highly aggressive tumour with distinct histopathalogical features, including high proliferation, necrosis and considerable neo-vascularisation (i.e., angiogenesis), leading to vessels that exhibit morphological abnormalities and ‘leakiness’ (Vajkoczy and Menger, 2000). It is generally accepted that the degree of angiogenesis is correlated to the malignancy of the tumour (Daumas-Duport *et al*, 1997a, 1997b). Therefore, assessment of the rate of tumour angiogenesis using specific biomarkers is potentially useful clinical information for determining the severity of the disease and selecting appropriate treatment modality.

Currently, there is a limited number of targeting ligands that have been utilised to non-invasively assess the degree of tumour angiogenesis. For GBM, these targeting moieties include the endogenous protein, vascular endothelial growth factor (VEGF), which binds to the vascular endothelial growth factor receptor 2 (VEGFR2) (Olsson *et al*, 2006), and Arg–Gly–Asp (RGD)-containing peptides that interact with endothelial cell adhesion molecules, such as *α*_v_*β*_3_ and *α*_v_*β*_5_ integrins (Varner and Cheresh 1996). Although these targeting moieties have shown promise in pre-clinical PET studies, some drawbacks, including heterogeneous target expression and nonspecific targeting to non-tumour vessels (Weibo *et al*, 2006; Schnell *et al*, 2009), remain unresolved. In our previous transcriptomic analyses of the laser-capture microdissected GBM vessels, the insulin-like growth factor-binding protein-7 (IGFBP7) was discovered as a highly upregulated selective biomarker of GBM vessels (Pen *et al*, 2007).

The IGFBP7, also known as IGFBP-related protein 1, mac25, TAF and angiomodulin, is a 31 kDa secreted protein that shares structural homology with a family of IGFBP-related proteins, which includes IGFBP1-6 (Kim *et al*, 1997). In contrast to IGFBP1-6 that displays high affinity for insulin-like growth factor (IGF), IGFBP7 is classified in the family subgroup showing low affinity for IGF (Kim *et al*, 1997). Several studies demonstrated that IGFBP7 is overexpressed in tumour blood vessels with little or no expression in normal blood vessels (Akaogi *et al*, 1996; St Croix *et al*, 2000; Pen *et al*, 2007), nor in the non-malignant angiogenic placental tissues (van Beijnum *et al*, 2006). IGFBP7 accumulates in the basement membrane of the tumour endothelium, where it can bind to collagen type II, IV and V, heparin sulphate proteoglycans and other cytokines (Nagakubo *et al*, 2003). Insights into mechanisms of IGFBP7 expression in the glioblastoma environment have come from *in vitro* experiments, which demonstrated that TGF-*β*1 secreted from glioblastoma tumour cells stimulate IGFBP7 production in human brain endothelial cells (HBECs) (Pen *et al*, 2008). IGFBP7 appears to increase the formation of capillary-like tubes by HBEC, suggesting a pro-angiogenic activity (Pen *et al*, 2007). Therefore, IGFBP7 is a selective and abundantly expressed biomarker of human GBM vessels that could be exploited for selective targeting of tumour vessels for imaging and therapeutic applications.

In this study, a llama anti-IGFBP7 single-domain antibody (sdAb) was developed and assessed in a mouse model of GBM for its ability to detect tumour vessels after *in vivo* administration. The sdAbs are small (13–15 kD) targeting molecules derived from the variable regions of heavy-chain antibodies from the camelid species (Hamers-Casterman *et al*, 1993). In contrast to IgG antibodies, sdAbs have fast pharmacokinetics due to small size, low nanomolar affinities when isolated from an immune library (Arbabi Ghahroudi *et al*, 1997) and can be easily engineered into a variety of antibody constructs (Conrath *et al*, 2001; Zhang *et al*, 2004). The anti-IGFBP7 sdAb developed in this study was demonstrated to bind specifically to both human and mouse GBM vessels in brain tissue sections and to tumour vessels after systemic injection in a mouse model of orthotopic glioblastoma. In addition, anti-IGFBP7 sdAb was shown to target PEGylated superparamagnetic nanoparticles (NPs) (T2-reducing MRI contrast agents) functionalised with the near-infrared probe Cy5.5 for optical detection, to the vessels of orthotopic brain tumour in mice, creating high tumour-contralateral side signal-to-noise ratio and selective tumour accumulation compared with other organs in prospective *in vivo* optical imaging. The results suggest that anti-IGFBP7 sdAb can be used to target appropriate contrast agents to abnormal tumour vasculature for non-invasive assessment of brain tumour angiogenesis using various imaging modalities.

## Methods

### Isolation of anti-IGFBP7-specific sdAbs from a llama immune phage display library

Recombinant human IGFBP7 protein was produced as described previously (Pen *et al*, 2008). A male llama (*Lama glama*) was injected subcutaneously with 100, 75, 75, 50 and 50 *μ*g IGFBP7 recombinant human protein on days 1, 21, 36, 50 and 64, respectively. Complete Freund's adjuvant (Sigma, Oakville, ON, Canada) was used for the primary immunisation and incomplete Freund's adjuvant was used for immunisations 2–4. Adjuvant was not used for the final immunisation. Total RNA was isolated from 2 × 10^7^ leukocytes using a QIAamp RNA Blood Mini Kit (Qiagen, Mississauga, ON, Canada). cDNA was synthesised using pd(N)_6_ primers. Three different sense primers (called J′ corresponding to the 5′-end of IgG), including MJ1 (5′-GCCCAGCCGGCCATGGCCSMKGTGCAGCTGGTGGAKTCTGGGGGA-3′), MJ2 (5′-CAGCCGGCCATGGCCCAGGTAAAGCTGGAGGAGTCTGGGGGA-3′) and MJ3 (5′-GCCCAGCCGGCCATGGCCCAGGCTCAGGTACAGCTGGTGGAGTCT-3′), and two anti-sense primers, corresponding to the C_H_2 domain DNA sequence, C_H_2 (5′-CGCCATCAAGGTACCAGTTGA-3′) and C_H_2_b_3 (5′-GGGGTACCTGTCATCCACGGACCAGCTGA-3′), were used to amplify the V_H_-C_H_1-hinge-C_H_2 region of conventional IgG or V_H_H-hinge-C_H_2. Amplified products of ∼600 bp from the primer combination J′-C_H_2 were extracted from a 1% agarose gel and the amplified products from primers J′-C_H_2_b_3 were PCR purified. In a second PCR, the two primers of MJ7BACK (5′-CATGTGTAGACTCGCGGCCCAGCCGGCCATGGCC-3′) and MJ8FOR (5′-CATGTGTAGATTCCTGGCCGGCCTGGCCTGAGGAGACGGTGACCTGG-3′) were used to introduce *Sfi*I restriction sites and to amplify the final sdAb fragments from the combined J′-C_H_2 and J′-C_H_2_b_3 amplified products. The final PCR product was digested with *Sfi*I and ligated into pMED1 (Arbabi-Ghahroudi *et al*, 2009), which was previously digested with the same restriction enzymes, and transformed into *E. coli* TG1 (New England Biolabs, Pickering, ON, Canada) by electroporation. A library size of 2 × 10^7^ was constructed and its complexity was determined by sequencing ∼30 randomly picked up colonies. Phage antibodies were rescued from the library with helper phage M13KO7 (New England Biolabs) and purified as described in Doyle *et al* (2008).

The llama immune phage display library was panned against purified IGFBP7. The VHHs recognising IGFBP7 were enriched by four consecutive rounds of *in vitro* selection. After each selection, the IGFBP7-specific phages were eluted with 100 mM triethylamine (pH 10.0) and immediately neutralised with 1 M Tris-HCl, pH 7.5. Exponentially growing TG1 cells were infected with the eluted phages followed by superinfection with M13KO7 helper phages. Finally, phages were amplified in a 50-ml baffled flask (2YT-Amp-Kan) overnight. After four rounds of panning, the eluted phages were used to infect exponentially growing *E. coli* TG1 cells. Individual colonies were grown, phage-rescued, and amplified phages were used in phage ELISA experiment.

For phage ELISA, wells of a 96-well plate were coated overnight with 5 *μ*g ml^–1^ IGFBP7 protein and then blocked with 1% casein for 2 h at 37°C. Phage (100 *μ*l) from individual clones were added to the pre-blocked wells and incubated for 1 h. Phage ELISA was performed using the Detection Module Recombinant Phage Antibody System (GE Healthcare, Baie d’Urfe, QC, Canada), and positive phage clones were sequenced. Sequencing results showed that the panning enriched mainly two clones with distinct sequences.

### Expression of anti-IGFBP7 sdAbs

DNA encoding the two positive clones was cloned into the *Bbs*I and *Bam*HI sites of a periplasmic expression vector, pSJF2, which added a c-Myc detection tag and a 5 × His purification tag at the C-terminus of the sdAbs. Anti-IGFBP7 sdAbs were expressed periplasmically and purified by IMAC as described previously (Abulrob *et al*, 2005).

### SPR analysis

Anti-IGFBP7 sdAbs were evaluated by surface plasmon resonance (SPR) using a Biacore 3000 (GE Healthcare) as described previously (Iqbal *et al*, 2010). Briefly, ∼1400 RU of recombinant human IGFBP7 (R&D Systems, Minneapolis, MN, USA) and 900 RU of ovalbumin (Sigma) as a reference protein were immobilised on a CM5 sensor chip using the amine-coupling kit supplied by the manufacturer. All binding studies were carried out at 25°C in 10 mM HEPES, pH 7.4 containing 150 mM NaCl, 3 mM EDTA and 0.005% surfactant P20. The flow rate used was 40 *μ*l min^–1^. The IGFBP7 surface was regenerated thorough washing with the running buffer. Data were analysed with BIAevaluation 4.1 software (GE Healthcare).

### Orthotopic brain tumour model

All animal procedures were approved by the NRC-IBS Animal Care Committee and were in strict compliance with the recommendations of the Canadian Council of Animal Care. Brain tumours were generated in nude CD-1 mice (male, 6–8 weeks old) by intracranial implantation of tumour-generating glioblastoma cell line, U87MG.EGFRvIII, carrying deletion mutant of EGFR (EGFRvIII) cells as described previously (Iqbal *et al*, 2010).

### Tissue preparation

Frozen human brain tumour tissues were obtained from Dr Garnette Sutherland (Foothills Hospital, Calgary, AB, Canada) with approval by both Foothills Hospital and NRC Research Ethics Boards. Human and mouse brain tissues were sectioned on a cryostat (Jung CM3000; Leica, Richmond Hill, ON, Canada) at 10 *μ*m thickness, and then mounted on Superfrost Plus microscope slides (Fisher Scientific, Nepean, ON, Canada).

### Immunohistochemisty

Frozen human or mouse brain tumour tissue sections were fixed in methanol at room temperature (RT). Slides were rinsed with 0.2 M PBS (pH 7.3), followed by blocking with 5% goat serum containing 0.1% Triton-X in PBS. Slides were then incubated with anti-IGFBP7 sdAb 4.43 (1 : 100 of a 1 mg ml^–1^ solution) for 3 h at RT, washed and then incubated with mouse monoclonal anti-c-myc primary (1 : 500) antibody for 1 h. Sections were washed five times before incubation with secondary antibody, goat anti-mouse alexa 647 (1 : 500; Molecular Probes, Eugene, OR, USA) for 1 h at RT. To stain blood vessels, human GBM tissue slides were incubated with Ulex europaeus I agglutinin (ULEX) (1 : 20; Vector Laboratories, Burlington, ON, Canada) for 3 min at RT, whereas mouse GBM tumour sections were incubated with rat anti-mouse CD31 primary antibody for 1 h at RT followed by goat anti-rat alexa 568 secondary antibody (1 : 300) for 1 h at RT. All slides were then washed and then cover slipped using DAKO fluorescent mounting (Dako, Burlington, ON, Canada) media containing Hoechst (1 : 1000; Sigma). In control slides, the anti-IGFBP7 sdAb was omitted.

### Synthesis of iron oxide PEGylated NPs

To synthesise 10 nm Fe_3_O_4_ NPs, 2 mmol of iron acetylacetonate, 10 mmol of 1, 2-hexadecanediol, 6 mmol of oleic acid, 6 mmol of oleylamine and 20 ml of phenyl ether were mixed and magnetically stirred in a three-neck 100 ml glass flask under nitrogen protection. The mixture was heated to 200°C for 30 min and, the temperature then was raised to 265°C and maintained for another 30 min. The mixture was then cooled to RT and ethanol was added to precipitate the product and the solid phase was obtained by centrifugation. The final product, 10 nm NPs as determined by transmission electron microscopy, was separated and purified from non-reacted residues by dissolving/precipitating cycles using a hexane/ethanol solvent pair. The final solution was prepared by re-dispersing the Fe_3_O_4_ wet solid into hexane. The powder form was obtained by drying the wet solid in vacuum. For PEGylation of the NPs, 40 mg of magnetic NPs were dissolved into 50 ml hexane and 1 ml oleylamine. The mixture was sonicated until the NPs were totally solubilised. The pH was set at 9 and 100 mg carboxy-amino-PEG (MW: 3000 Da) (Creative PEGs works, Winston Salem, NC, USA) was added and heated to reflux for 2 h. The supernatant was removed and a grey solid was washed three to five times with hexane and dried under nitrogen.

### Bioconjugation of sdAbs to PEGylated NPs

PEGylated NPs were solubilised in PBS buffer (PBS, 0.5 mM EDTA, pH 7.4) such that the final concentration of iron was 1 mg Fe ml^–1^. Cy5.5-NHS ester was added to the NP solution at 50-fold molar excess and reacted for 1 h at RT. Unreacted dye was removed using 50 kDa Amicon ultra centrifugal units (Millipore, Billerica, MA, USA).

A 500-fold molar excess anti-IGFBP7 sdAb 4.43 was reconstituted in MES buffer (0.1 M MES, 0.5 M NaCl, pH 5.5). To introduce NHS-ester functionality on the sdAb, Sulfo-NHS and EDC were added at 180- and 70-fold molar excess, respectively, and reacted for 30 min at RT. Subsequently, EDC was removed by centrifugation using 3 kDa Amicon columns. Cy5.5-labelled PEGylated NPs were added to the antibody solution and reacted at RT for 4–6 h while mixing. Unreacted sdAbs were removed by purification in 100 kDa Amicon columns. Anti-IGFBP7 sdAb-NP-Cy5.5 formulation was resuspended in PBS (0.5 mM EDTA, pH 7.4). Protein absorbance at 280 nm, after subtracting for background absorbance from PEGylated NPs alone, indicated ∼50 sdAbs attached per PEGylated NP. Dynamic light scattering on both PEGylated NPs and anti-IGFBP7 sdAb-PEGylated NPs was done using a Zetasizer nano (Malvern Instruments, Worcestershire, UK). The dynamic light scattering experiments were carried out at 23°C on samples dispersed (1 : 100) in milliQ water. The following parameters were used for data analysis to reflect solid particles in water: material refractive index of 1.59, a dispersant refractive index of 1.33 and a dispersant viscosity of 0.9308. Size distribution was determined by intensity-weighted data analysis.

### Pharmacokinetic analysis

Non-targeted Cy5.5-labelled PEGylated NPs were injected via tail vein in normal CD-1 mice. Blood samples of 25 *μ*l were collected by creating a small nick in the tail vein followed by collection of blood in a heparanised tube. Blood samples were collected at multiple time points at 5 min, 30 min, 1, 1.5, 2, 4, 24 and 48 h after injection. Samples were analysed for labelled NPs using a fluorescent plate reader with excitation at 670 nm and emission at 690 nm and compared with a standard curve of a range of concentrations of the labelled NPs diluted in blood. Pharmacokinetic parameters were calculated with a two-compartment, IV-bolus model using the WinNonlin pharmacokinetic software 5.2 (Pharsight Corporation, Mountain View, CA, USA).

### *In vivo* near-infrared optical imaging

Anti-IGFBP7 sdAb 4.43 was labelled with Cy5.5 succinimidyl ester using methods recommended by the manufacturer (GE Healthcare). Labelling was optimised to achieve a dye/antibody ratio of one. Anti-IGFBP7 sdAb-Cy5.5 (50 *μ*g for IGFBP7sdAb 4.43 or 50 *μ*g negative control (NC) sdAb) were injected via the tail vein in mice bearing 10-day-old U87MG.EGFRvIII brain tumours. Additionally, a competition experiment was also performed in which 100-fold excess concentration of unlabelled anti-IGFBP7 sdAb was injected intravenously 10 min before the injection of 50 *μ*g IGFBP7sdAb-Cy5.5. In other experiments brain tumour-bearing mice were injected with anti-IGFBP7-sdAb NP-Cy5.5 or non-targeted NP-Cy5.5 (10 mg Fe per kg body weight) via the tail vein. Animals were subjected to *in vivo* imaging studies using a small-animal time-domain eXplore Optix MX2 pre-clinical imager (Advanced Research Technologies, Montreal, QC, Canada) as described previously (Abulrob *et al*, 2007, 2008). Average fluorescence concentration data from ROIs placed around the tumour region were subsequently analysed using the OptiView software package (Advanced Research Technologies). At the end of each experiment, mice were injected with 40 *μ*g of fluorescein-labelled tomato lectin, 10 min before killing to stain blood vessels. Animals were then perfused with heparanised saline and organs and brain tumours were excised and imaged *ex vivo. Ex vivo* organs were analysed by placing an ROI around each organ and determining the total fluorescence concentration per gram tissue.

### Fluorescent microscopy

Coronal sections (50 *μ*m) were produced using a Vibratome sectioning instrument (Ted Pella, Redding, CA, USA). Tissue sections were mounted on Superfrost Plus microscope slides (Fisher Scientific, Nepean, ON, Canada) using mounting media containing 2 *μ*g ml^–1^ Hoescht nuclei stain (Sigma). Sections were then visualised under an Olympus 1X 81 inverted motorised microscopes (Olympus, Markham, ON, Canada). *InVivo* and ImagePro 6.2 software (Olympus, Markham, ON, Canada) were used to acquire and analyse images. Some sections were alternatively stained with hematoxylin (0.1% hematoxylin, 5% alum, 0.02% sodium iodate, 0.1% citric acid) and 1% eosin Y.

### Statistical analysis

All data are reported as mean±s.e.m. and the differences between groups were determined using two-way ANOVA followed by the Bonferoni *post hoc* test. Differences greater than *P*<0.05 were considered significant.

## Results

### Binding by SPR

**Figure 1:**
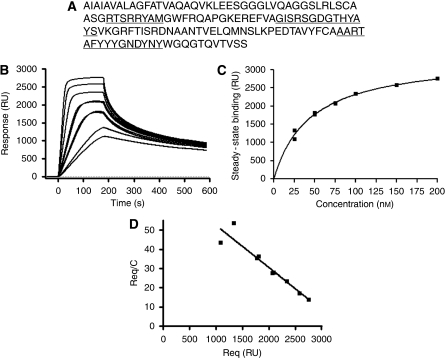
Protein sequence and surface plasmon resonance analysis of the anti-IGFBP7 sdAb 4.43. (**A**) Protein sequence of anti-IGFBP7 sdAb 4.43; CDR1, CDR2 and CDR3 are underlined. (**B**) Sensogram overlay showing 4.43 monomer binding to immobilised IGFBP7 at concentrations of 25, 25, 50, 50, 75, 75, 100 and 200 nM. (**C**) Fitting of equilibrium binding data to a steady-state affinity model. (**D**) Scatchard plots of the equilibrium binding data.

Based on phage ELISA results (data not shown), two clones showed binding to IGFBP7, clones 4.43 and 4.46. In SPR analyses, clone 4.43 showed strong binding to antigen but the affinity of clone 4.46 for IGFBP7 was in the low micromolar range (data not shown). As a result, only clone 4.43 was selected for further analysis both *in vitro* and *in vivo* studies. The binding data for 25 nM 4.43 (Figure 1A) fit quite well to a 1 : 1 model, giving a *k*_a_ of 6.6 × 10^4^ m s^−1^, a *k*_d_ of 1.3 × 10^−3^ s^−1^ and a *K*_D_ of 20 nM. However, at higher 4.43 concentrations the 1 : 1 fitting was poor with the dissociation phases being clearly biphasic (Figure 1A). Nevertheless, the equilibrium binding data showed good fitting to a steady state affinity model (Figure 1B) and gave a linear Scatchard plot (Figure 1C) from which *K*_D_'s of 40 and 50 nM, respectively, were derived. Inexplicably, the observed *R*_max_ for the equilibrium data was several fold higher that the theoretical *R*_max_ for a 1 : 1 interaction in which monovalent sdAb binds to an immobilised antigen without repeating sequences, an observation that may compromise the kinetic and affinity constant calculations.

### Immunofluorescence staining of mouse and human GBM tissues with anti-IGFBP7 sdAb

**Figure 2:**
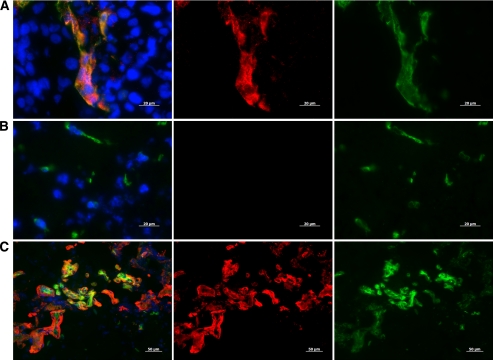
Representative immunofluorescence images demonstrating IGFBP7 immunoreactivity detected with the anti-IGFBP7 sdAb 4.43 in tissue sections of the (**A**) mouse orthotopic GBM, (**B**) contralateral healthy mouse brain and (**C**) human GBM. IGFBP7 immunoreactivity is shown in red (middle panels); the staining for the endothelium-specific markers, CD31 and UEA1, for mouse and human tissues, respectively, is shown in green (right panels) and cell nuclei stained with DAPI are shown in blue in overlay images (left panels).

The ability of anti-IGFBP7 sdAb to detect IGFBP7 expression in mouse and human GBM tissue was evaluated by immunofluorescence (Figure 2). In brain sections of GBM-bearing mice, IGFBP7 immunoreactivity detected by anti-IGFBP7 sdAb (clone 4.43) was enhanced and detected specifically in the tumours (Figure 2A), but not in normal brain (Figure 2B), and colocalised with the immunoreactivity against the vascular antigen, CD31 (Figure 2A). No IGFBP7 expression was observed in non-tumour brain vessels or in non-vascular cells. In surgically removed human GBM tissue, IGFBP7 immunoreactivity (Figure 2C, middle panel) colocalised with highly abnormal tumour vasculature stained with Ulex europeaus agglutinin I (UEA I) (Figure 2C, right panel), shown previously to selectively bind to carbohydrates of human brain vessels (Holthofer *et al*, 1982). These results indicate that anti-IGFBP7 sdAb detects its target, IGFBP7, in both mouse and human GBM tissue sections *ex vivo.* This analysis confirms our previous observation of a selective vascular upregulation of IGFBP7 in human GBM (Pen *et al*, 2007) and demonstrates that mouse vessels ingrowing into orthotopically implanted human GBM (U87MG-EGFRvIII) are selectively induced by the tumour microenvironment to overexpress IGFBP7. This mouse model is thus suitable for *in vivo* assessment of tumour targeting/imaging using anti-IGFBP7 sdAb.

### *In vivo* biodistribution of anti-IGFBP7 sdAbs

**Figure 3:**
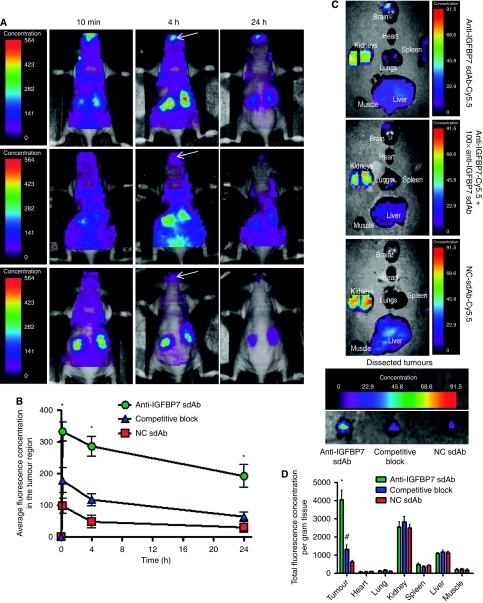
*In vivo* optical imaging of mice bearing orthotopic glioblastoma tumours after intravenous (i.v.) administration of anti-IGFBP7 sdAb labelled with the near-infrared fluorescent probe, Cy5.5. (**A**) Dorsal whole body *in vivo* optical images of mice bearing U87MG.EGFRvIII brain tumours at indicated time points after i.v. injection of 50 *μ*g of either anti-IGFBP7 sdAb-Cy5.5 (upper panels), 100 × unlabelled anti-IGFBP7 sdAb followed by anti-IGFBP7 sdAb-Cy5.5 (competitive block, middle panels) or NC sdAb-Cy5.5 (lower panels) (arrows indicate the location of the brain tumour). (**B**) Graph illustrating changes in the average fluorescence concentration determined from an ROI in the brain tumour region *in vivo* at indicated times after the i.v. injection of sdAbs-Cy5.5. (**C**) Optical images of organs *ex vivo* and dissected brain tumours 24 h after injection of sdAbs-Cy5.5 (**D**) Graph illustrating the total fluorescence concentration per gram tissue in various organs and dissected tumours imaged *ex vivo* 24 h after the injection of sdAbs-Cy5.5. In **B** and **D**, the data are expressed as mean±s.e.m. for *n*=5 animals. ^*^Indicates significant difference between anti-IGFBP7 sdAb and both competitively blocked anti-IGFBP7 sdAb and NC sdAb (*P*<0.01). ^#^Indicates significant difference between competitively blocked anti-IGFBP7-sdAb and NC sdAb (*P*<0.05).

The *in vivo* and *ex vivo* biodistribution analyses of the systemically injected anti-IGFBP7 sdAb ‘tagged’ with the near-infrared dye Cy5.5 alone or in combination with the 100-fold excess of unlabelled anti-IGFBP7-sdAb or NC sdAb-Cy5.5 were performed in mice bearing a 10-day-old U87MG.EGFRvIII orthotopic GBM tumour using prospective *in vivo* optical imaging (Figure 3A). The anti-IGFBP7 sdAb-Cy5.5 homed to the brain tumour as early as 10 min after injection (Figure 3A, upper panels; Figure 3C), with high signal in the tumour persisting up to 24 h after injection. In contrast, brain tumour signal *in vivo* was reduced at 4 and 24 h when animals were co-injected with the excess unlabelled anti-IGFBP7 sdAb (Figure 3A, middle panels; Figure 3B). NC sdAb-Cy5.5 (Figure 3A, lower panels; Figure 3B) was barely detectable in the brain tumour at any time. At 24 h after injection, brain tumour signal for the anti-IGFBP7 sdAb-Cy5.5 was three- and six-fold higher compared with competitively blocked anti-IGFBP7 sdAb-Cy5.5 and NC sdAb-Cy5.5, respectively (Figure 3B). As sdAbs have a blood half-lives in the range of 20–30 min and are completely cleared from the blood within the first few hours (Iqbal *et al*, 2010) after injection, the high tumour signal detected at 4 and 24 h with anti-IGFBP7 sdAb-Cy5.5, which was competitively attenuated in the presence of the excess of unlabelled anti-IGFBP7 sdAb, indicates retention of the antibody in the tumour due to its binding to the tumour-expressed antigen.

Organs (liver, kidney, spleen, lung, heart, brain and muscle) and extracted tumour biodistribution (Figure 3C) assessed by *ex-vivo* organ imaging 24 h after injection confirmed the selective brain tumour targeting of anti-IGFBP7 sdAb, but not NC sdAb. Similar to the *in vivo* data, the *ex-vivo* data at 24 h demonstrated a three- and six-fold increase in brain tumour signal for the anti-IGFBP7 sdAb compared with the competitively blocked anti-IGFBP7 sdAb-Cy5.5 and NC sdAb-Cy5.5, respectively (Figure 3D). No optical signals were detected in the contralateral normal brain. Fluorescence lifetime analysis confirmed that 90% of the signal in the brain tumour originated from sdAb linked to Cy5.5 (*τ*=2.1 ns), in contrast to a virtual absence of the free Cy5.5 fluorescence lifetime (*τ*=1.1 ns) (data not shown). Both sdAbs (anti-IGFBP7 and NC) gave a high kidney signal, which is the main elimination route for sdAbs, whose size of 13 kD is below kidney filtration cutoff.

### Fluorescent microscopy confirmation of injected anti-IGFBP7 sdAb-Cy5.5 in brain tumours

**Figure 4:**
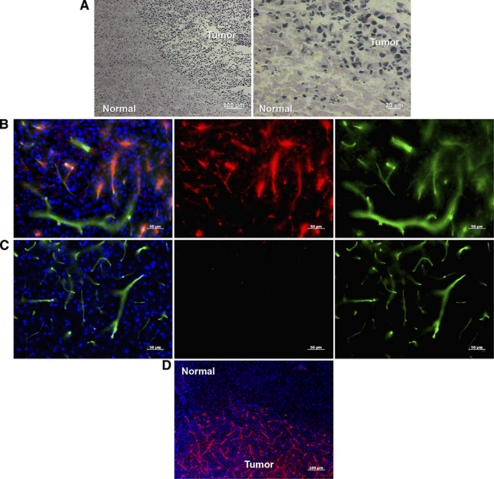
Fluorescent and light microscopic images of mouse orthotopic GBM tumour sections obtained 24 h after intravenous (i.v.) injection of 100 *μ*g anti-IGFBP7 sdAb-Cy5.5. (**A**) Sections of brain tumour stained with hematoxylin and eosin staining showing brain tumour boundaries from the ipsilatelar healthy brain tissue (left panel, low magnification; right panel, high magnification). Higher magnification fluorescent images of tumour centre (**B**) and contralateral healthy brain region (**C**) showing colocalisation of the systemically injected anti-IGFBP7 sdAb-Cy5.5 (red; middle panels) and brain vessels stained with the 40 *μ*g fluorescein tomato lectin (green; right panels) injected i.v. 10 min before killing, and their composite overlay images with nuclei stained with DAPI (blue; left panels). Scale bar: 50 *μ*m. (**D**) Lower magnification image of the tumour and normal brain illustrating that injected anti-IGFBP7 sdAb-Cy5.5 (red) decorates selectively a majority of tumour vessels 24 h after i.v. injection. Scale bar: 100 *μ*m.

U87MG brain tumours grew in a spherical shape that was easily distinguishable from the surrounding health brain, when visualised by hematoxylin and eosin staining (Figure 4A). At higher magnification, hematoxylin and eosin staining clearly illustrates the demarcation of the increased cellularity of the tumour *vs* the normal brain regions (Figure 4A, right panel).

At the end of imaging protocol (24 h after injection), brain sections were analysed by fluorescent microscopy to examine localisation of injected Cy5.5-labelled sdAbs. In contrast to NC-sdAb-Cy5.5 fluorescence, which could not be detected in either tumour or healthy brain tissue sections (data not shown), anti-IGFBP7 sdAb-Cy5.5 fluorescence (red) was selectively localised in brain tumour vessels stained with injected lectin (Figure 4B), but not in healthy brain tissue vessels (Figure 4C). In lower-magnification images the fluorescence of injected anti-IGFBP7 sdAb-Cy5.5 was present throughout the tumour in virtually all vessels (Figure 4D). These results confirm that the increased tumour optical signal observed in *in vivo* optical imaging resulted from the selective ‘homing’ of anti-IGFBP7 sdAb to abnormal, IGFBP-expressing brain tumour vessels.

### Characterisation of iron oxide NPs

**Figure 5:**
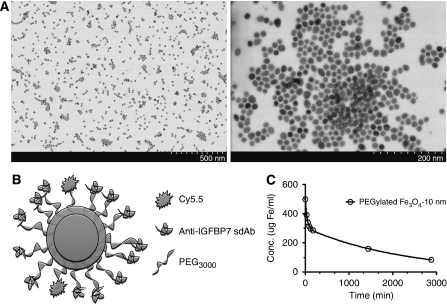
(**A**) Representative transmission electron microscopy image of NPs at two magnifications. (**B**) Schematic diagram of the anti-IGFBP7 sdAb conjugated to a PEGylated Fe_3_O_4_ NP and labelled with Cy5.5. (**C**) Graph of 10 nm core PEGylated NP-Cy5.5 concentration in serum over time, fitted to a two-compartment, IV-bolus model (WinNonLin 5.2).

The Fe_3_O_4_-based NPs (10 nm core) were monodisperse and uniform, as shown in representative transmission electron microscopy images in Figure 5A. The NPs were coated with hundreds of primary amine-PEG_3000_ molecules to serve as attachment moieties for Cy5.5 and multiple sdAbs (Figure 5B). The presence of amine-PEG_3000_ was confirmed using fluorescamine, a non-fluorescent agent that fluoresces when it reacts with primary amines (Udenfriend *et al*, 1972) (data not shown). PEGylation produced NPs that were stable and water soluble. Dynamic light scattering of the PEGylated NP revealed a hydrodynamic diameter of 99 nm with a polydispersity index (PDI) of 0.2. PDI is a measure of particle size distribution and values <0.2 can be considered to be narrowly distributed. Bioconjugation of sdAb to the PEGylated NP increased the average particle size to 124 nm, while maintaining a PDI of 0.18. *In vivo*, PEGylation resulted in NPs with a circulation half-life of approximately 24 h (Figure 5C).

### Biodistribution and tumour targeting of anti-IGFBP7sdAb-PEGylated NPs-Cy5.5 *in vivo*

**Figure 6:**
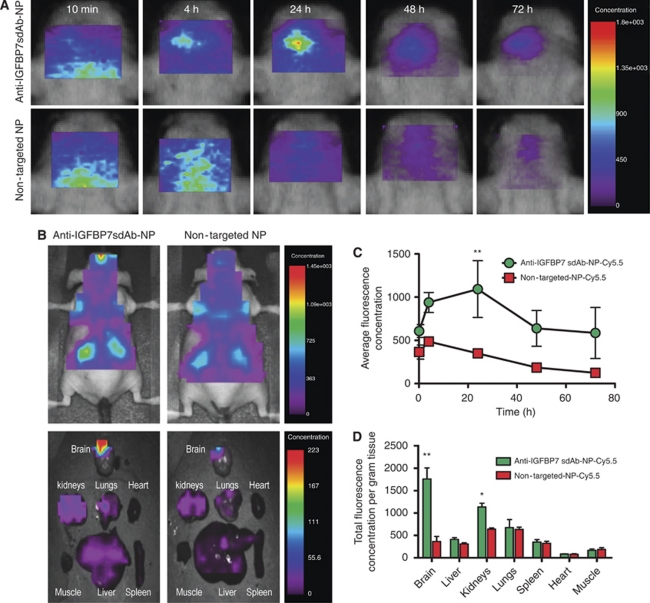
*In vivo* optical imaging of the biodistribution of non-targeted and anti-IGFBP7 sdAb-targeted Fe_3_O_4_ nanoparticles labelled with Cy5.5 injected at 10 mg Fe kg^–1^ intravenous (i.v.) in mice bearing orthotopic glioblastoma tumours. (**A**) *In vivo* images of the head indicated time points after the injection of anti-IGFBP7-targeted (upper panels) and non-targeted (lower panels) NPs. (**B**) *In vivo* optical images of whole animal body (upper panels) 72 h after i.v. injection of anti-IGFBP7-targeted and non-targeted NPs and corresponding *ex vivo* organ images after animal killing by perfusion (bottom panels). (**C**) Graph showing changes of the average fluorescence concentration in the brain tumour region *in vivo* at indicated times after the injection of either anti-IGFBP7 sdAb-targeted or non-targeted-NPs-Cy5.5. (**D**) Graph illustrating the total fluorescence concentration per gram tissue in organs imaged *ex vivo* 72 h after the injection of either anti-IGFBP7 sdAb-targeted or non-targeted-NPs-Cy5.5. In **C** and **D**, data are expressed as mean±s.e.m. for *n*=5 animals. ^**^Indicates significant difference between anti-IGFBP7 sdAb-NP-Cy5.5 and non-targeted-NP-Cy5.5 (*P*<0.001). ^*^Indicates significant difference between the anti-IGFBP7 sdAb-NP-Cy5.5 and non-targeted-NP-Cy5.5 (*P*<0.01).

The biodistribution of anti-IGFBP7 sdAb-PEGylated NPs-Cy5.5 and non-targeted PEGylated NPs-Cy5.5 after systemic injection was examined by *in vivo* imaging in mice bearing 10-day-old orthotopic glioblastoma tumours. Whereas a progressive increase in optical signal peaking at 24 h after injection was observed in the brain tumour region in animals injected with the anti-IFGBP7 sdAb-PEGylated NPs-Cy5.5 (Figure 6A, upper panels, Figure 6C), no specific accumulation of non-targeted NPs was observed in the head ROI (Figure 6A, bottom panels; Figure 6C. Anti-IGFBP7 sdAb-PEGylated NPs-Cy5.5 fluorescence signal in the tumour region diminished after 24 h (Figure 6A, upper panels, Figure 6C). However, when the signal-to-noise ratio was determined by dividing the average fluorescence concentration values in the tumour region with that in the contralateral healthy brain region, a steady increase of the signal, peaking at 72 h was observed in the tumour region (data not shown). The whole-body scans (Figure 6B, upper panels) and *ex vivo* organ imaging (Figure 6B; bottom panels; Figure 6D) at 72 h after injection demonstrated that the brain tumour signal of anti-IGFBP7 sdAb-PEGylated NPs-Cy5.5 is the highest compared with all other organs. Similarly, at 72 h, *ex vivo*-measured fluorescence in the brain was five-fold higher in mice injected with anti-IGFBP7sdAb-targeted compared with non-targeted PEGylated NPs.

### Fluorescent microscopy confirmation of injected anti-IGFBP7 sdAb-PEGylated NPs-Cy5.5 in brain tumours

**Figure 7:**
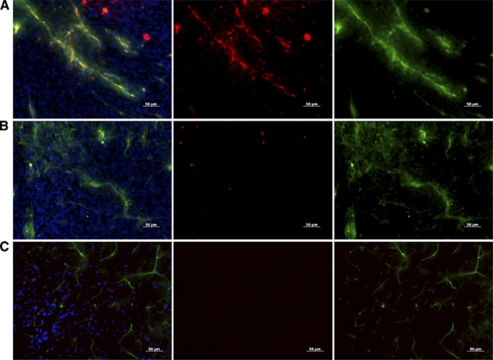
Fluorescent microscopic images of mouse GBM tumour sections obtained 72 h after intravenous injection of 10 mg Fe kg^–1^ of either anti-IGFBP7 sdAb-targeted (**A**) or non-targeted (**B**) Fe_3_O_4_ nanoparticles labelled with Cy5.5 (red). (**C**) Contralateral healthy brain region of animals injected with anti-IGFBP7-sdAb-targeted NPs. Mice were also injected with 40 *μ*g of FITC-labelled tomato lectin, 10 min before killing to stain blood vessels *in vivo*. Lectin staining (green; right panels) colocalises with the Cy5.5 signal (red; middle panels) of NPs in overlay images (left panels; cell nuclei-blue) only in the tumour region of animals injected with anti-IGFBP7-targeted NPs. Scale bar: 50 *μ*m.

Sections from GBM tumour-bearing mice receiving anti-IGFBP7 sdAb-PEGylated NPs-Cy5.5 demonstrated Cy5.5 fluorescence (red) in the tumour region, colocalising with the tomato-lectin-stained vessels (green) (Figure 7A) compared with virtual absence of Cy5.5 fluorescence in tumour sections from animals injected with non-targeted PEGylated NPs-Cy5.5 (Figure 7B). The contralateral healthy brain did not show any measurable Cy5.5 signal in animals injected with either anti-IGFBP7 sdAb-targeted (Figure 7C) or non-targeted PEGylated NPs-Cy5.5 (data not shown).

## Discussion

The aim of the study was to generate novel targeting moieties against brain tumour vasculature useful in brain tumour imaging. IGFBP7 was confirmed in this study as a selective vascular target that is overexpressed in both human GBM and in an orthotopic mouse model of GBM. The anti-IGFBP7 sdAb was then raised and assessed for use as molecular imaging agent/diagnostic for GBM. The sdAb format was chosen due to its small size (15 kDa), fast clearance kinetics and moderate affinity for its targets, characteristics advantageous in molecular imaging applications (Lin *et al*, 2010). After panning an immunised library for binders, an sdAb with moderate affinity (*K*_d_ in low nanomolar range) for the IGFBP7 target was selected. In contrast to control sdAb, anti-IGFBP7 sdAb fluorescently tagged for detection was found to bind specifically to both human and mouse GBM tumour vessels *ex vivo* and to brain tumour vessels in mice after injection *in vivo*, creating high signal-to-noise ratio in orthotopic brain tumours easily detectable by *in vivo* optical imaging. In addition, we show that the anti-IGFBP7 sdAb could be utilised to target bimodal optical-MRI contrast agent based on Fe_3_O_4_ PEGylated NPs labelled with Cy5.5. Using *in vivo* optical imaging, brain tumour selective targeting in mice was demonstrated for the anti-IGFBP7 sdAb-PEGylated-NPs-Cy5.5, in contrast to a non-targeted-PEGylated-NPs-Cy5.5. The results of the study suggest that anti-IGFBP7 sdAbs and anti-IGFBP7 sdAb-PEGylated-NPs are both promising molecular imaging agents for targeting brain tumour vessels with potential for clinical translation.

The VEGFR2 is another target highly expressed on tumour endothelial cells (Ferrara 2004) and exploited for developing molecular imaging strategies for tumours. The targeting moiety, ^64^Cu-labelled vascular endothelial growth factor 121 (VEGF_121_) has been developed for non-invasive PET imaging of VEGFR expression in small animals (Cai *et al*, 2006). Similar to anti-IGFBP7 sdAb, VEGF_121_ has a low nanomolar affinity and fast clearance from the body due to its small size (25 kDa) (Goncalves *et al*, 2005). In pre-clinical PET studies, VEGFR expression was shown to be heterogeneous in GBM xenografts depending on the size of the tumour (Cai *et al*, 2006). Another well-studied marker of GBM tumour vessels explored for non-invasive imaging of tumour vessels (Schottelius *et al*, 2009) is the *α*_v_*β*_3_ integrin, against which several RGD containing peptide binders were developed. In a recent study in which [^18^F]galacto-RGD peptide was used for PET imaging assessment of *α*_v_*β*_3_ integrin expression in patients with GBM (Schnell *et al*, 2009), it was demonstrated that GBMs had very heterogeneous tracer uptake, which, however, correlated well with immunohistochemically determined *α*_v_*β*_3_ expression. In contrast to IGFBP7, *α*_v_*β*_3_ integrin expression is also associated with non-tumour angiogenic vessels and tumour cells (Cairns *et al*, 2003). Anti-IGFBP7 sdAb have advantages compared with other predominantly peptide-based strategies, including high specificity to the target, while retaining appropriate pharmacokinetic characteristics for imaging applications, including PET; further studies comparing anti-IGFBP7 sdAb with other brain tumour-vessel targeting molecular imaging agents in PET studies are warranted. In addition to GBMs, high vascular expression of IGFBP7 has been shown in some peripheral tumours, including oesophagus, lung and stomach cancers (Akaogi *et al*, 1996).

An added benefit of the anti-IGFBP7 sdAb-targeted bimodal NPs developed in this study is the possibility of using MRI to non-invasively assess tumour angiogenesis. MRI is a widely available modality with high spatial resolution for *in vivo* imaging, whereas optical imaging currently remains a useful pre-clinical tool. In this study, anti-IGFBP7 sdAb PEGylated NPs displayed specific targeting to brain tumours compared with non-targeted PEGylated NPs. The anti-IGFBP7 sdAb-targeted PEGylated NPs also displayed a greater level of targeting compared with the anti-IGFBP7 sdAb alone. This effect may be attributed to an increased avidity for the IGFBP7 target, as a result of the multivalent nature of the NPs (i.e., multiple sdAbs bound to each NP). Although not analysed in this study, many reports have described an increase in binding avidity (up to four orders of magnitude) for multivalent targeted NPs (Hong *et al*, 2007; Iqbal *et al*, 2010; Tassa *et al*, 2010). An additional feature of PEGylated NPs is their longer circulation half-life (days) *vs* non-PEGylated NPs (hours) or sdAbs alone (mins). The level of PEGylation on the NPs can be adjusted and the pharmacokinetics of the NPs can be further tailored (Lim *et al*, 2008). For clinical use, a targeted NP that can achieve a high signal to background ratio, followed by complete elimination from the body would be ideal.

Despite many therapeutic advances in the field, most patients with GBM still have poor prognosis, with a life expectancy of 12–15 months (Strupp *et al*, 2005). In contrast, low-grade gliomas (WHO grade II) show little or no neo-vascularisation and much better prognosis, with a median survival ranging from 4–16.7 years depending on the age and histology of the patient (Olson *et al*, 2000). However, malignant transformation is always a risk for low-grade tumours, such that 13–86% of tumours initially diagnosed as low grade recur at a higher histological grade (Barker *et al*, 1997). The malignancy of the tumour is highly correlated to the degree of angiogenesis (Daumas-Duport *et al*, 1997a, 1997b) and the progression from low to higher tumour grades is commonly referred to as an ‘angiogenic switch’ (Moserle *et al*, 2009). It is reasonable to argue that treatments should be applied early, either when the angiogenic switch occurs or to prevent the angiogenic switch from happening in the first place. To achieve information on such a switch, however, there is a need for accurate markers of tumour angiogenesis. As lower-grade glioma tissue sections do not express IGFBP7 (D Stanimirovic, unpublished observations), anti-IGFBP7 sdAb may feasibly represent a useful follow-up molecular imaging tool in determining when the ‘angiogenic switch’ has been turned on for low-grade gliomas. By providing information on the degree of tumour angiogenesis and related clinical aggressiveness, the anti-IGFBP7 sdAb could also be used to assess/monitor efficacy of anti-angiogenic or other anti-tumour treatments, and thus improve the clinical management of brain tumours. The high expression and accessible nature of the IGFBP7 target in GBM vessels, combined with virtually non-existent expression in normal vessels, in combination with demonstrated versatility and good *in vivo* targeting characteristics of anti-IGFBP7 sdAb, indicate that linking of this antibody to the appropriate optical, PET or MRI contrast agents could enable *in vivo* imaging assessment of the degree of angiogenesis in gliomas.

## References

[bib1] Abulrob A, Brunette E, Slinn J, Baumann E, Stanimirovic D (2007) *In vivo* time domain optical imaging of renal ischemia-reperfusion injury: discrimination based on fluorescence lifetime. Mol Imaging 6: 304–314 18092515

[bib2] Abulrob A, Brunette E, Slinn J, Baumann E, Stanimirovic D (2008) Dynamic analysis of the blood-brain barrier disruption in experimental stroke using time domain *in vivo* fluorescence imaging. Mol Imaging 7: 248–262 19123995

[bib3] Abulrob A, Sprong H, Van Bergen en Henegouwen P, Stanimirovic D (2005) The blood-brain barrier transmigrating single domain antibody: mechanisms of transport and antigenic epitopes in human brain endothelial cells. J Neurochem 95: 1201–1214 16271053 10.1111/j.1471-4159.2005.03463.x

[bib4] Akaogi K, Okabe Y, Sato J, Nagashima Y, Yasumitsu H, Sugahara K, Miyazaki K (1996) Specific accumulation of tumor-derived adhesion factor in tumor blood vessels and in capillary tube-like structures of cultured vascular endothelial cells. Proc Natl Acad Sci USA 93: 8384–8389 8710880 10.1073/pnas.93.16.8384 PMC38680

[bib5] Arbabi Ghahroudi M, Desmyter A, Wyns L, Hamers R, Muyldermans S (1997) Selection and identification of single domain antibody fragments from camel heavy-chain antibodies. FEBS Lett 414: 521–526 9323027 10.1016/s0014-5793(97)01062-4

[bib6] Arbabi-Ghahroudi M, To R, Gaudette N, Hirama T, Ding W, MacKenzie R, Tanha J (2009) Aggregation-resistant VHs selected by *in vitro* evolution tend to have disulfide-bonded loops and acidic isoelectric points. PEDS 22: 59–66 19033278 10.1093/protein/gzn071

[bib7] Barker FG, Chang SM, Huhn SL, Davis RL, Gutin PH, McDermott MW, Wilson CB, Prados MD (1997) Age and the risk of anaplasia in magnetic resonance-non-enhancing supratentorial cerebral tumors. Cancer 80: 936–941 9307194

[bib8] Cai W, Chen K, Mohamedali KA, Cao Q, Gambhir SS, Rosenblum MG, Chen X (2006) PET of vascular endothelial growth factor receptor expression. J Nucl Med 47: 2048–2056 17138749

[bib9] Cairns RA, Khokha R, Hill RP (2003) Molecular mechanisms of tumor invasion and metastasis: an integrated view. Curr Mol Med 3: 659–671 14601640 10.2174/1566524033479447

[bib10] Conrath KE, Lauwereys M, Galleni M, Matagne A, Frere JM, Kinne J, Wyns L, Muylderman S (2001) Beta-lactamase inhibitors derived from single-domain antibody fragments elicited in the camelidae. Antimicrob Agents Chemother 45: 2807–2812 11557473 10.1128/AAC.45.10.2807-2812.2001 PMC90735

[bib11] Daumas-Duport C, Tucker ML, Kolles H, Cervera P, Beuvon F, Varlet P, Udo N, Koziak M, Chodkiewicz JP (1997a) Oligodendrogliomas. Part II: A new grading system based on morphological and imaging criteria. J Neurooncol 34: 61–78 9210053 10.1023/a:1005759220434

[bib12] Daumas-Duport C, Varlet P, Tucker ML, Beuvon F, Cervera P, Chodkiewicz JP (1997b) Oligodendrogliomas. Part I: Patterns of growth, histological diagnosis, clinical and imaging correlations: a study of 153 cases. J Neurooncol 34: 37–59 9210052 10.1023/a:1005707203596

[bib13] Doyle PJ, Arbabi-Ghahroudi M, Gaudette N, Furzer G, Savard ME, Gleddie S, McLean MD, Mackenzie CR, Hall JC (2008) Cloning, expression, and characterization of a single-domain antibody fragment with affinity for 15-acetyl-deoxynivalenol. Mol Immunol 45: 3703–3713 18632156 10.1016/j.molimm.2008.06.005

[bib14] Ferrara N (2004) Vascular endothelial growth factor: basic science and clinical progress. Endocr Rev 25: 581–611 15294883 10.1210/er.2003-0027

[bib15] Goncalves M, Estieu-Gionnet K, Berthelot T, Lain G, Bayle M, Canron X, Betz N, Bikfalvi A, Deleris G (2005) Design, synthesis, and evaluation of original carriers for targeting vascular endothelial growth factor receptor interactions. Pharm Res 22: 1411–1421 16078152 10.1007/s11095-005-5265-9

[bib16] Hamers-Casterman C, Atarhouch T, Muyldermans S, Robinson G, Hamers C, Songa EB, Bendahman N, Hamers R (1993) Naturally occurring antibodies devoid of light chains. Nature 363: 446–448 8502296 10.1038/363446a0

[bib17] Holthofer H, Virtanen I, Kariniemi AL, Hormia M, Linder E, Miettinen A (1982) Ulex europaeus I lectin as a marker for vascular endothelium in human tissues. Lab Invest 47: 60–66 6177923

[bib18] Hong S, Leroueil PR, Majoros IJ, Orr BG, Baker JR, Banaszak Holl MM (2007) The binding avidity of a nanoparticle-based multivalent targeted drug delivery platform. Chem Biol 14: 107–115 17254956 10.1016/j.chembiol.2006.11.015

[bib19] Iqbal U, Trojahn U, Albaghdadi H, Zhang J, O’Connor-McCourt M, Stanimirovic D, Tomanek B, Sutherland G, Abulrob A (2010) Kinetic analysis of novel mono- and multivalent VHH-fragments and their application for molecular imaging of brain tumors. Br J Pharm 160(4): 1016–1028 10.1111/j.1476-5381.2010.00742.x PMC2936006 20590596

[bib20] Kim HS, Nagalla SR, Oh Y, Wilson E, Roberts CT, Rosenfeld RG (1997) Identification of a family of low-affinity insulin-like growth factors binding proteins (IGFBPs): characterization of connective tissue growth factor as a member of the IGFBP superfamily. Proc Natl Acad Sci USA 94: 12981–12986 9371786 10.1073/pnas.94.24.12981 PMC24249

[bib21] Lim J, Guo Y, Rostollan CL, Stanfield J, Hseih JT, Sun X, Simanek EE (2008) The role of the size and number of polyethylene glycol chains in the biodistribution and tumor localization of triazine dendrimers. Mol Pharm 5: 540–547 18672950 10.1021/mp8000292

[bib22] Lin X, Xie J, Chen X (2010) Protein-based tumor molecular imaging probes. Amino Acids, doi:10.1007/500726-010-0545-2 10.1007/s00726-010-0545-z PMC3617487 20232092

[bib23] Moserle L, Amadori A, Indraccolo S (2009) The angiogenic switch: implications in the regulation of tumor dormancy. Curr Mol Med 9: 935–941 19925406 10.2174/156652409789712800

[bib24] Nagakubo D, Murai T, Tanaka T, Usui T, Matsumoto M, Sekiguchi K, Miyasaka M (2003) A high endothelial venule secretory protein, mac25/angiomodulin, intereacts with multiple high endothelial venule-associated molecules including chemokines. J Immunol 171: 553–561 12847218 10.4049/jimmunol.171.2.553

[bib25] Olson JD, Riedel E, DeAngelis LM (2000) Long term outcome of low-grade oligodendroglioma and mixed glioma. Neurology 54: 1442–1448 10751254 10.1212/wnl.54.7.1442

[bib26] Olsson AK, Dimberg A, Kreuger J, Claesson-Welsh L (2006) VEGF receptor signalling – in control of vascular function. Nat Rev Mol Cell Biol 7: 359–371 16633338 10.1038/nrm1911

[bib27] Pen A, Moreno MJ, Durocher Y, Deb-Rinker P, Stanimirovic DB (2008) Glioblastoma-secreted factors induce IGFPB7 and angiogenesis by modulating Smad-2-dependent TGF-*β* signalling. Oncogene 27: 6834–6844 18711401 10.1038/onc.2008.287

[bib28] Pen A, Moreno MJ, Martin J, Stanimirovic DB (2007) Molecular markers of extracellular matrix remodelling in glioblastoma vessels: microarray study of laser-captured glioblastoma vessels. Glia 15: 559–572 10.1002/glia.20481 17266141

[bib29] Schnell O, Krebs B, Carlsen J, Miederer I, Goetz C, Goldbrunner RH, Wester HJ, Haubner R, Popperl G, Holtmannspotter M, Kretzschmar HA, Kessler H, Tonn JC, Schwaigner M, Beer AJ (2009) Imaging of integrin alpha(v)beta(3) expression in patients with malignant glioma by 18F Galacto-RGD positron emission tomography. Neuro Oncol 6: 861–870 10.1215/15228517-2009-024 PMC2802406 19401596

[bib30] Schottelius M, Laufer B, Kessler H, Wester HJ (2009) Ligands for mapping alpha_v_beta_3_ expression *in vivo*. Acc Chem Res 42: 969–980 19489579 10.1021/ar800243b

[bib31] St Croix B, Rago C, Velculescu V, Traverso G, Romans KE, Montgomery E, LaI A, Riggins GJ, Lengauer C, Vogelstein B, Kinzler KW (2000) Genes expressed in human tumor endothelium. Science 18: 1121–1122 10.1126/science.289.5482.1197 10947988

[bib32] Strupp R, Mason WP, van den Bent MJ, Weller M, Fisher B, Taphoorn MJ, Belanger K, Brandes AA, Marosi C, Bogdahn U, Curshmann J, Janzer RC, Ludwin SK, Gorlia T, Allgeier A, Lacombe D, Cairncross JG, Eisenhauer E, Mirimanoff RO (2005) Radiotherapy plus concomitant and adjuvant temozolomide for glioblastoma. N Engl J Med 352: 987–996 15758009 10.1056/NEJMoa043330

[bib33] Tassa C, Duffner JL, Lewis TA, Weissleder R, Schreiber SL, Koehler AN, Shaw SY (2010) Binding affinity and kinetic analysis of targeted small molecule-modified nanoparticles. Bioconjug Chem 21: 14–19 20028085 10.1021/bc900438a PMC2902264

[bib34] Udenfriend S, Stein S, Bohlen P, Dairman W, Leimgruber W, Weigele M (1972) Fluorescamine: a reagent for assay of amino acids, peptides, proteins, and primary amines in the picomole range. Science 178: 871–872 5085985 10.1126/science.178.4063.871

[bib35] Vajkoczy P, Menger MD (2000) Vascular microenvironment in gliomas. J Neurooncol 50: 99–108 11245285 10.1023/a:1006474832189

[bib36] van Beijnum JR, Dings RP, van der Linden E, Zwaans BM, Ramaekers FC, Mayo KH, Griffioen AW (2006) Gene expression of tumor angiogenesis dissected: specific targeting of colon cancer angiogenic vasculture. Blood 108: 2339–2348 16794251 10.1182/blood-2006-02-004291

[bib37] Varner JA, Cheresh DA (1996) Integrins and cancer. Curr Opin Cell Biol 8: 724–730 8939661 10.1016/s0955-0674(96)80115-3

[bib38] Weibo C, Kai C, Khalid A, Khalid M, Cao Q, Gambhir S, Rosenblum MG, Chen X (2006) PET of vascular endothelial growth factor receptor expression. J Nucl Med 47: 2048–2056 17138749

[bib39] Zhang J, Tanha J, Hirama T, Khieu NH, To R, Tong-Sevinc H, Stone E, Brisson JR, MacKenzie CR (2004) Pentamerization of single-domain antibodies from phage libraries: a novel strategy for the rapid generation of high-avidity antibody reagents. J Mol Biol 335: 49–56 14659739 10.1016/j.jmb.2003.09.034

